# Real-world case studies for a process-aware IDS

**DOI:** 10.1186/s42162-025-00545-1

**Published:** 2025-06-17

**Authors:** Verena Menzel, Johann Hurink, Anne Remke

**Affiliations:** 1 EEMCS-MOR, University of Twente, P.O. Box 217, 7500 AE Enschede, Netherlands; 2 Safety-critical systems group, University of Münster, Einsteinstraße 62, 48149 Münster, Germany

**Keywords:** Intrusion detection system, Smart grids, Process-aware

## Abstract

The transition to sustainable energy increasingly relies on robust communication infrastructure to monitor, control, and optimize energy distribution. Supervisory Control and Data Acquisition (SCADA) networks manage these processes, transmitting sensor data and control commands. However, integrating (smart) communication systems into an ageing existing communication infrastructure introduces vulnerabilities to cyber-attacks, such as false data injection and man-in-the-middle attacks. Although recent advancements in Intrusion Detection Systems (IDS) for SCADA networks show potential in detecting domain-specific threats, testing has largely been confined to simulations due to the nature of critical infrastructure. This paper presents two real-world case studies using actual grid data, where a process-aware IDS solution is tailored to specific network topologies. The result effectively detects various cyber-attacks, including those targeting critical devices like transformers. This work marks a crucial step toward practical deployment, emphasizing the need for a gradual transition from simulation to real-world validation to ensure the safety and reliability of critical grid infrastructure.

## Introduction

In the transition of the global energy sector towards more sustainable and decentralized sources, the modernization of communication infrastructure becomes a critical challenge. As more diverse communication devices, such as smart meters, electric vehicle charging stations, and remote controls for Medium Voltage substations, are integrated into the electricity grid, the need for interoperable communication systems becomes increasingly critical. For effective planning, reliable real-time data from various grid locations is needed when managing fluctuating and to a larger extend uncontrollable renewable energy production and demand. Currently, Supervisory Control and Data Acquisition (SCADA) systems support the continuous management of power system operations. However, from a cyber-security perspective, SCADA communication infrastructure is often outdated as common protocols like Modbus, DNP3, and IEC-104 lack systematic cyber-security features, making these systems particularly vulnerable to cyber-attacks [1, 2].

In recent years, attackers already have increasingly exploited weaknesses in SCADA communication protocols and infrastructure [3, 4]. Notable incidents include the Stuxnet attack on process automation in 2010 [5], the Duqu and Flame malware in 2011 and 2012 [6, 7], and the 2015 attack on the Ukrainian power grid using BlackEnergy malware [8]. These incidents underscore the urgent need for specialized intrusion detection systems (IDS) designed to address the unique security challenges of the energy sector [9].

SCADA systems operate under specific conditions, including limited computational capabilities in field devices and strict real-time constraints. Traditional IT security solutions are often unsuitable in this context, as they may exceed the processing capacity of field devices [10]. Additionally, their analysis and decision-making processes can introduce delays, making them incompatible with the time-sensitive nature of SCADA operations. To date, only a few SCADA-specific security approaches exist, most of which involve deep-packet-inspection (DPI) and the analysis of packet content in the context of the state of the system [11–13]. Developing and testing security solutions for SCADA systems is inherently challenging due to several factors. First, testing within critical large-scale infrastructure poses significant risks, as any maintenance and disruption can have severe consequences. Second, as mentioned above, SCADA systems often integrate diverse legacy components, each with unique Operational Technology (OT) constraints, such as strict real-time requirements and limited computational resources. Lastly, regulatory and compliance requirements, such as the worldwide standard IEC 62443 [14], the North American NERC CIP [15], NIST CSF 2.0 [16] or NIST SP 800-82 [17] as well as the European NIS 2 directive [18], govern security measures to protect the systems, but may also complicate the research development and evaluation process.

In response to these challenges, Chromik et al. [19–23] introduced a local, process-aware IDS for the power distribution system, bringing together the physical and the cyber layer and aimed at detecting anomalies within the SCADA network. This IDS has since then been expanded to include information from neighbouring field stations, enabling the detection of synchronized attacks on multiple field stations simultaneously [24]. Additional requirements based on power flow equations for larger topologies have also been incorporated to enhance detection capabilities [25]. Unlike traditional approaches, which focus on higher-level network traffic analysis, the process-aware IDS developed by Chromik et al. [19–23] evaluates the physical consistency of communicated sensor data directly at field stations, allowing for a more granular assessment. This approach facilitates faster communication with the grid operator, particularly in cases where the system state may be distorted, helping to prevent erroneous decisions. To understand the installation and deployment process of the IDS on distributed hardware and to identify potential hardware delays, recently the process-aware IDS has been tested on a distributed Raspberry Pi cluster [26]. Furthermore, communication challenges and attacks targeting the IDS itself were addressed and a mitigation strategy using rumour-spreading algorithms was proposed [27]. While these developments give insight into the hardware side of building an actual physical prototype that can be tested at real field stations, they still utilized artificial simulation data. More recently, the concept has also been extended to multi-energy systems, using DEMKit [28, 29] as the foundation for the testbed instead of the co-simulation framework mosaik [30] broadening its applicability [31].

This paper addresses the mitigation of the system to real-world data, investigating the effects of measurement inaccuracies and synchronization problems between measurement points that are geographically spread. Furthermore, the paper highlights the importance of fine-tuning the IDS to strike an optimal balance between false positives and false negatives, allowing the operator to decide where to focus their attention. Note that at this stage, we do not test the IDS on a real deployed system but rather a prototype implementation with real life network measurements. We advocate that the tests presented in this paper are a necessary intermediate step between the simulation scenarios conducted so far and building an actual physical prototype that can be tested at real field stations.

Where the original approach of the group around Chromik includes the analysis of commands transmitted over the SCADA network, this paper focuses solely on sensor readings. A reason for this is that the two real-world datasets used in this paper lack command data, implying that incorporating commands would have required guessing them and simulating the corresponding grid responses. Instead, we prioritize the use of real-world sensor data without relying on additional simulation, which aligns with the primary focus of this work.

Note that our proposed IDS solution is primarily tailored for Distribution System Operators (DSOs), focusing on the grid level between Medium Voltage (MV) and Low Voltage (LV) stations, as well as local feeders. This level is critical for the operational security of DSOs and aligns with the datasets used in our case studies. Following this line, the contributions of this paper are as follows:

- We introduce an adapted and novel methodology for defining and organizing requirements for the IDS as presented in [19–23].
- We propose a method for effective threshold calibration concerning the sensitivity of the IDS, balancing between false positives and false negatives.
- We demonstrate the applicability of this approach through an in-depth analysis of two real-world case studies, utilizing genuine operational data and infrastructure information: one focusing on the DSO perspective with the transition between the LV and MV grids, and another on a rural area.
- We present an adaptable and flexible IDS implementation that can be tailored towards different grid topologies and customized rule sets.
- We showcase the effectiveness of the IDS with a virtually distributed evaluation consisting of six scenarios, all based on real-world grid data and authentic infrastructure configurations.

The paper is organized as follows: Section "Related work" presents relevant related work and proposes the specific aspects of SCADA attacks and the attack model considered in this paper. Furthermore, the original process-aware IDS is summarized. Section "Real-world case studies " presents the two real-world case studies and in Section "Methodological extension of the IDS", we discuss the adaptions of the IDS requirements for the two case studies and the topology characteristics and highlight important design decisions taken during the implementation. In Section "Evaluation" the two case studies are evaluated using three different scenarios each. Next to this in, in Section "Discussion" an impact analysis of the used tolerance values is conducted, thereby considering the sensitivity and specificity of the IDS, and the results of the evaluation and the proposed approach. The paper is concluded in Section "Conclusion".

## Related work

In this Section, we describe SCADA systems in general (see Section "SCADA architecture and security" ) and the work on dedicated security in this area. We also provide the attack model used in this paper, which is a combined man-in-the-middle and false-data injection attack, performed at remote field stations (see Section "Attack model" ). Furthermore, we analyse the line of work presented by Chromik et al. [19–23] which proposes a process-aware IDS (see Section "Background on process-aware IDS" ) and serves as foundation for the IDS used in this paper.

### SCADA architecture and security

SCADA networks play an important role in the control of industrial control systems, such as energy distribution, oil and gas production and distribution, as well as water cleaning and distribution. Traditionally, SCADA systems were built as monolithic systems without security in mind. However, with the ongoing digitization and an increasing need for remote control, SCADA systems have evolved from distributed architectures to networked and even IoT-based systems. In the following, we provide a short overview of SCADA systems and their vulnerabilities. For a detailed discussion, we refer e.g. to [32].

**Fig. 1:**
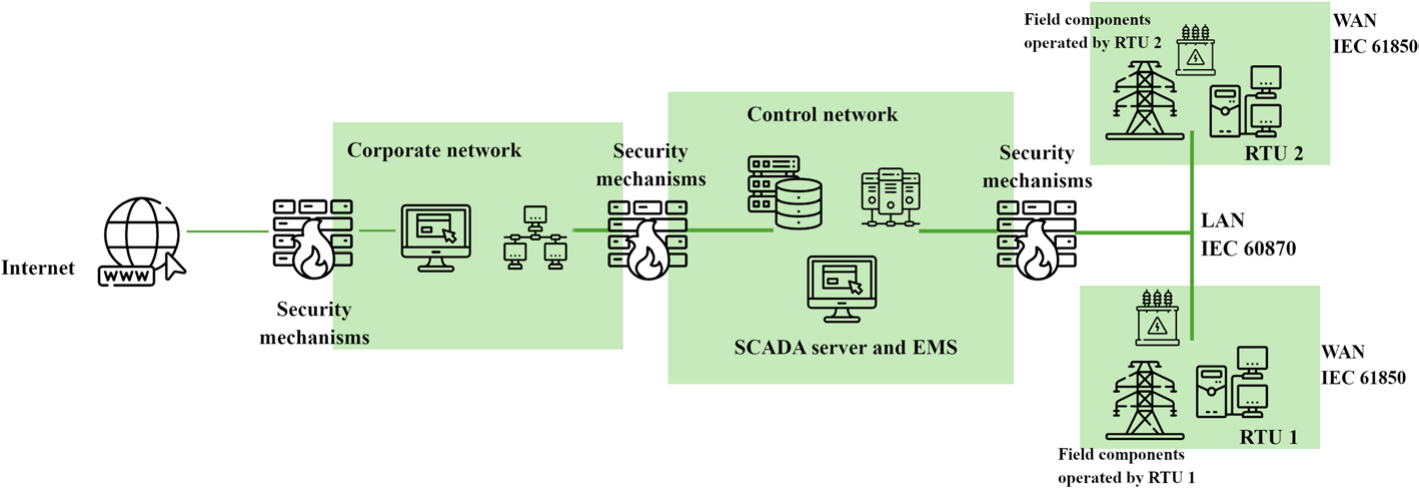
Networked SCADA architecture (control network) in the context of a corporate network and field components. (Adapted from [33].)

The networked SCADA architecture shown in Fig. 1 consists of interconnected layers: a corporate network, a control network, and field components.

The corporate network connects to the Internet and implements security mechanisms (e.g., firewalls) to prevent unauthorized access. The control network houses the SCADA server and the EMS which are responsible for managing industrial processes and ensuring secure communication between components. The field layer consists of Remote Terminal Units (RTUs) that control field devices like substations and meters. Originally, SCADA communication relied on proprietary protocols, such as Modbus RTU, RP-570, Profibus, and Conitel [34]. Today, there is a shift toward open-source protocols, including Modbus TCP/IP, DNP3, IEC 61850, and IEC 60870 [7].

- Modbus TCP/IP works as a client-server architecture via Ethernet. It lacks encryption and relies on the lower layer protocols for checksum protection.
- DPN3 is an open-source protocol, which allows flexible and secure communication between components. It features error-checking, data-timestamping, and generic data types [35]. However, it lacks thorough authentication, which may facilitate unauthorized access.
- The international standard IEC 60870 facilitates control e.g. between RTUs at substations and their field devices, respectively.
- IEC 60870-104 is a variant which is commonly used for communication between a control station and its substation via a standard TCP/IP network. It does not define access passwords, authentication or encryption [36]. Note that this opens the door for attackers e.g. to change the value of IEC 104 packets, to insert spoofed messages into the network, to perform distributed denial of service attacks (DDoS), and to intercept the transmitted data [37].

Despite the isolation of the control network from the Internet, which is often the case, it is important to recognize that security by separation alone is not sufficient. In practice, smart grid communication is typically air-gapped and protected through access control mechanisms and N-1 criterion to ensure system resilience. However, the underlying communication protocols remain largely unchanged in terms of security capabilities. These protocols, as discussed above, were not designed with modern cybersecurity requirements in mind. Consequently, they lack essential features such as encryption, strong authentication, and robust integrity protection. This creates a risk in scenarios where an attacker manages to bypass perimeter defences, particularly through insider threats or compromised devices within the trusted network.

Open platform communication (OPC) [38] is one of the most important communication standards for IoT and Industry 4.0. It standardizes a client-server architecture, which provides access to machines, devices, and other parts of the system in a platform-independent way. Specifically, OPC UA standardizes the data exchange between machines and systems, incorporating security features such as encryption, message signing, packet sequencing, and user authentication. OPC UA also uses a certificate exchange for controlling which client connects to which server. However, it has been shown that many UPC UA artifacts have one (or more) security issues. [39] illustrates that also in an OPC UA communication, attackers can steal credentials, eavesdrop on exchanged messages, manipulate the physical process through sensor values and actuator commands, and prevent the detection of anomalies, if implemented incorrectly. This illustrates that common SCADA protocols typically lack elaborate cyber-security mechanisms, making them susceptible to exploitation. In the context of intrusion detection, specific approaches for OPC UA traffic are already being researched e.g. [40].

IoT-based SCADA systems are often integrated into cloud environments and offer increased flexibility and availability. However, they also introduce additional security risks, such as unauthorized access to sensitive data and control systems. Although IoT-based SCADA solutions are gaining traction globally, their adoption remains limited, particularly in Europe, where operators remain cautious about transitioning to cloud-based architectures.

Following the taxonomy presented in [41], security measures for industrial environments can be classified into four main categories: network monitoring (including network-based intrusion detection systems (NIDS)), endpoint protection, event management, and physics-based monitoring. Since this work focuses on an IDS that incorporates physics-based monitoring, we first provide an overview of IDS and then delve into specific physics-based approaches tailored to electricity grids.

To protect SCADA networks against cyber-attacks, three primary types of IDS exist: (i) specification-based, (ii) behaviour-based, and (iii) signature-based approaches. Specification-based IDS define permitted actions, issuing alerts for deviations, yet may overlook attacks following allowed traffic sequences [42, 43]. This limitation can be mitigated by involving packet content or order inspection, as shown in [44–47]. Behaviour-based approaches like [48–51], also sometimes called anomaly-based approaches, compare the current power grid state to an ideal state, often causing false positives and failing to detect stealthy attacks [52, 53]. Signature-based approaches, such as [52], rely on historical attack comparisons, demanding periodic database updates and facing challenges in detecting *zero-day* attacks. Often, the signature-based attacks also include learning-based approaches, such as [54–56].

As indicated in Figure 1, most cyber-security measures in the energy context are usually implemented around the corporate network and the control network. These measures focus primarily on the prevention and detection of cyber threats. For instance, firewalls are commonly used to block uncontrolled access, helping to prevent Distributed Denial of Service (DDoS) attacks, phishing attempts, and the import of malware via Trojan horses. Furthermore, password authentication prevents unauthorized access in general and the specification of user roles helps to limit access to critical infrastructure even further. Additional security measures also include appropriate backup strategies and patching used software [57].

For the sake of clarity and focus in this paper, we limit our discussion to these preventive and detective measures. Other important aspects of cybersecurity, such as response, recovery, physical security, and system resilience, are not investigated further. While such general cyber-security measures are implemented by most power operators by now, the field equipment often remains unprotected [58]. In combination with the vulnerabilities of the communication protocols described above, unprotected field stations are becoming a lucrative target for attackers [59].

Notably, most existing IDS originate from traditional ICT and are not adapted to the energy context. However, recently physics-based detection approaches like [11–13] are researched. Further, [60] presents an application of a physics-informed neural network used for anomaly detection, while [61] uses machine-learning specifically in the detection of false data injection (FDI) attacks. For a more in-depth review of physic-based anomaly detection approaches and the usage of AI, we refer to [62].

State estimation (SE) is another crucial option dedicated to power grids to detect manipulations and malfunctions. In SE, the collected measurements are transmitted to a central or distributed entity (often the SCADA server), which then calculates the system state [63, 64]. This calculated state is subsequently compared to the existing system state based on this data [65, 66]. However, SE relies on the assumption that the input data gathered from the field devices and communicated over the SCADA network, is correct. FDI attacks can therefore hugely impact the results of SE [67, 68]. Correspondingly, countermeasures against FDI in the context of state estimation are studied both for power grids and also other cyber-physical systems [69–75].

### Attack model

As described in the previous section, SCADA communication infrastructure is particularly vulnerable to attackers exploiting weaknesses in protocols, such as the lack of authentication.

The attack model in this paper assumes that attackers are familiar with the communication protocols in use and understand common SCADA architectures. They exploit protocol vulnerabilities to infiltrate the control network or field stations, either directly through missing authentication or via Trojan horse methods. This infiltration allows them to eavesdrop, intercept, and manipulate communication packets in a *man-in-the-middle attack*. Additionally, attackers can impersonate legitimate parties, an attack known as *spoofing*. Specifically, in the context of electricity grids, manipulating sensor readings during a *man-in-the-middle attack* is referred to as a *false-data-injection attack* (FDI).

In this paper we focus on the following three attack scenarios:

1. Attackers gain access to a field station communicating with the control network using IEC 60870-104 over LAN or WAN (*man-in-the-middle attack*).
2. Attackers intercept communication to learn packet formats and patterns.
3. Attackers manipulate intercepted packets by altering (sensor) data and forwarding them to the SCADA server (*false-data-injection*). These manipulations may involve manually altering individual messages or replaying entire historical datasets.

Attackers with domain knowledge might be aware of existing security measures like bad-data detection (BDD) and they may estimate or know BDD thresholds and limit their manipulations to avoid detection. The goal of such attacks is to present a false view to the Energy Management System or state estimation, which may remain undetected for an extended period. This deception may lead to the operator issuing incorrect and harmful commands, which could result in partial or widespread blackouts. For example, in 2015, a power outage occurred in the Northern part of the Netherlands including Amsterdam and Schiphol Airport, after a technical defect was followed by human error. In that incident, an operator supposedly made an incorrect decision based on a misinterpretation of a visual observation, highlighting how relying on flawed or misleading information can lead to serious operational consequences [76].

Note that due to security features commonly added during practical deployment, such as access control and network segmentation, launching simultaneous and coordinated attacks at multiple locations is not necessarily trivial.

### Background on process-aware IDS

**Fig. 2:**
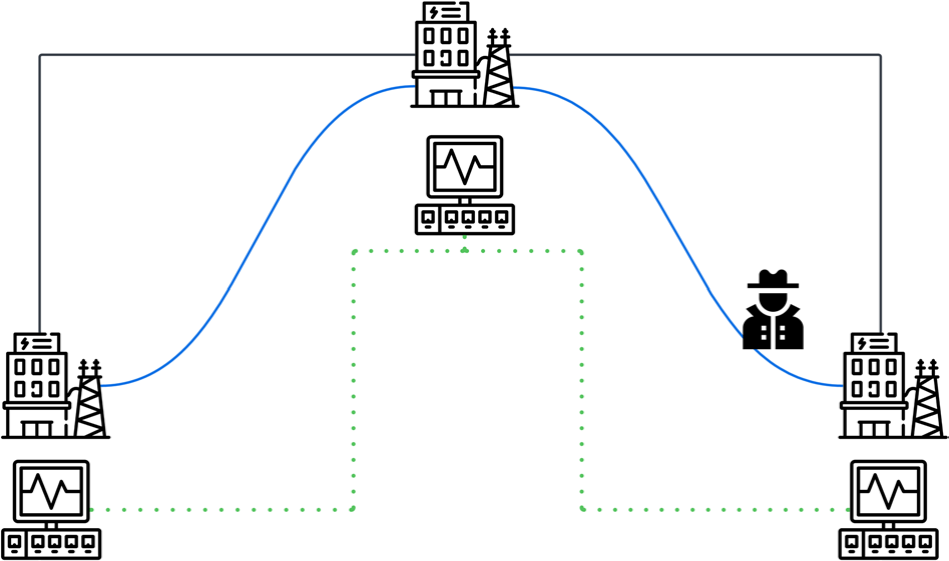
Conceptual overview over the IDS in case of three subgrids connected via power lines (black) and attacked SCADA network (blue). The additional IDS communication channels between the monitors is indicated in dotted green

We focus on the *process-aware IDS* approach, considered in [19, 20, 23], which performs deep-packet inspection locally at field stations by analysing SCADA network traffic. An overview of the approach is given in Figure 2. This IDS is designed as a distributed, multi-component system where local monitors evaluate incoming and outgoing packets at each substation against an internal model of the connected sensors and valves. If deviations in sensor measurements are detected, an alert is sent to the operator. Similarly, when a command could endanger equipment, such as e.g. exceeding the maximum current on a line, the system triggers a warning to prevent unsafe actions. This localized approach aims to enable real-time detection of and response to anomalies directly at each substation. For more details, we refer to [21, 22].

This process-aware IDS has been extended to provide a broader, more comprehensive view by incorporating data from neighbouring field stations [24, 25]. Neighbourhood monitors have been introduced to supervise the *border regions* between substations, evaluating data from connecting components like power lines. These neighbourhood monitors coordinate with local monitors to ensure that the values from both sides of a connection remain consistent. This additional layer of supervision allows for a more sophisticated analysis using e.g. Power Flow Equations to verify that the combined data from multiple substations presents a coherent and accurate picture of the grid. It is important to note that the IDS does not communicate directly over the standard SCADA network but instead passively monitors its traffic. All exchanges between the IDS monitors occur via separate, secure communication channels, bypassing the security vulnerabilities of the existing network. This is indicated as the green dotted lines in Fig. 2.

The concept of process-aware IDS has mostly been tested in simulation environments, as for example within the mosaik co-simulation framework [30]. One notable exception is [23], where the approach has been successfully tested at a power distribution substation in the Netherlands. The test indicated that the system had a very low miss rate and high precision.

To emulate the behaviour of multiple neighbouring substations, a distributed prototype on a Raspberry Pi cluster has been developed to evaluate the real-time capacities of the used hardware and the chosen communication protocol [26]. This line of work continued towards investigating true self-organization and protection in case of attacks against the distributed IDS, i.e., its local monitors, through integrating gossiping algorithms [27]. While these works represent important steps towards an actual physical prototype, they still rely on simulation data from the co-simulation framework mosaik [30]. Moreover, the approach assumed perfect availability of data in adequate quality at each substation, which may hold for simulation environments, but not necessarily in real electrical grids.

This paper addresses the lack of case studies with a larger amount of real-life data. It considers the testing the proposed IDS infrastructure in two settings for which real-world data is available. In the context of critical infrastructure it is important when developing security-realistic solutions, to start with smaller simulation data sets. Based on this, it is a necessary first step for conceptual ideas and algorithms to be developed. These smaller simulation datasets can be used to refine detection algorithms and assess baseline performance in a controlled environment. The next step is to progress to offline live data, as proposed in this paper, as it introduces real-world complexities such as data quality issues, missing time synchronization, and limited data availability into the system. Addressing these challenges is critical to ensure that the IDS performs reliably under realistic conditions. This layered process mitigates the risks associated with the direct deployment of new software in critical infrastructure to ensure safe operation and security of supply. Furthermore, it helps to avoid unintended consequences such as alert fatigue, where frequent false positives undermine the operator’s trust in the system.

## Real-world case studies

In this section, we present the two real-world case studies to which the process-aware IDS is applied: the first case study (see Section"Real-world case study 1 - DSO") features two connected MV stations and is therefore called the DSO Case Study and the second case study (see Section"Real-world case study 2 - rural area") features a LV feeder substation with five measured branches, including a farm, a house, and a big solar roof. Therefore, it is called the rural area case study. In the following, we describe the general setup and the existing topology for both case studies.

Note that in general, power system measurements serve different purposes and are collected by distinct systems. Protection data, metering values, and control signals are typically acquired through separate channels. In our two case studies presented below, we abstract from this complexity and assume the attacker can manipulate the measurements relevant to the operator’s decision-making, such as state estimation inputs and control data. This simplification allows us to focus on the potential impact of data manipulation without modelling the entire measurement infrastructure.

### Real-world case study 1 - DSO

**Fig. 3:**
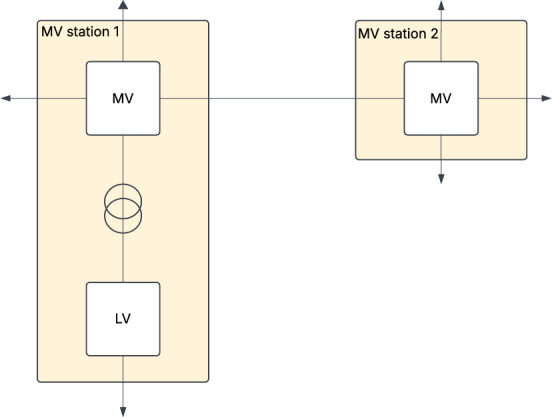
Schematic overview over the first real-world case study. Arrows indicate connection to other parts of the grid, which are not considered in this scenario

The first case study examines a power distribution network consisting of two MV stations and one LV measurement unit, recorded at the LV side of a transformer connected to one of the MV stations. An overview of the setup is given in Fig. 3

The monitoring system of the distribution system operator continuously collects data from both MV stations through the SCADA network. In general constant real-time monitoring is not always applied, however, the system can perform real-time checks and trigger alarms in case of global outages. The LV measurements in this setup are deployed temporarily to record the data for this case study. The operator regularly uses such measurements for diagnostics. These measurements are important in identifying power quality issues, such as high voltage during peak solar generation or voltage asymmetry due to uneven load distribution. In cases, they also may be used for fraud detection.

Legally, LV data is for the operator’s lowest level with direct measurement access. Household smart meter data is limited to consumption and billing purposes due to privacy regulations. As such LV station data forms an important asset for operational monitoring while maintaining compliance with privacy laws.

In principle, the LV side of the transformer should not be considered a separate node within the network. However, since those measurements were placed and recorded independently of the connected MV substation, we consider it a third location in the context of the IDS. The LV data contains three phased measurements for voltage and current, as well as noise measurements and the Total Harmonic Distortion (THD). THD is a measure of the distortion in an electrical signal caused by the presence of harmonics and is expressed as the ratio of the sum of all harmonic components to the fundamental frequency component. Therefore, it can be used as an indication for power quality.

For the two involved MV stations the data consists of single-phased current measurements for each power line connected to that field station as well as three-phased voltage measurements at the field station. All these data is also equipped with timestamps. They are recorded with a fixed 10-minute time step, whereby the MV stations starts at 12:00, the LV data starts at 12:02. This time resolution suffices for evaluation purposes, as it aligns with the temporal granularity used by the DSO for monitoring and operating the grid. As a result, minor fluctuations or manipulations in the communicated grid data that occur on shorter time scales are not typically acted upon by the operator.

### Real-world case study 2 - rural area

**Fig. 4:**
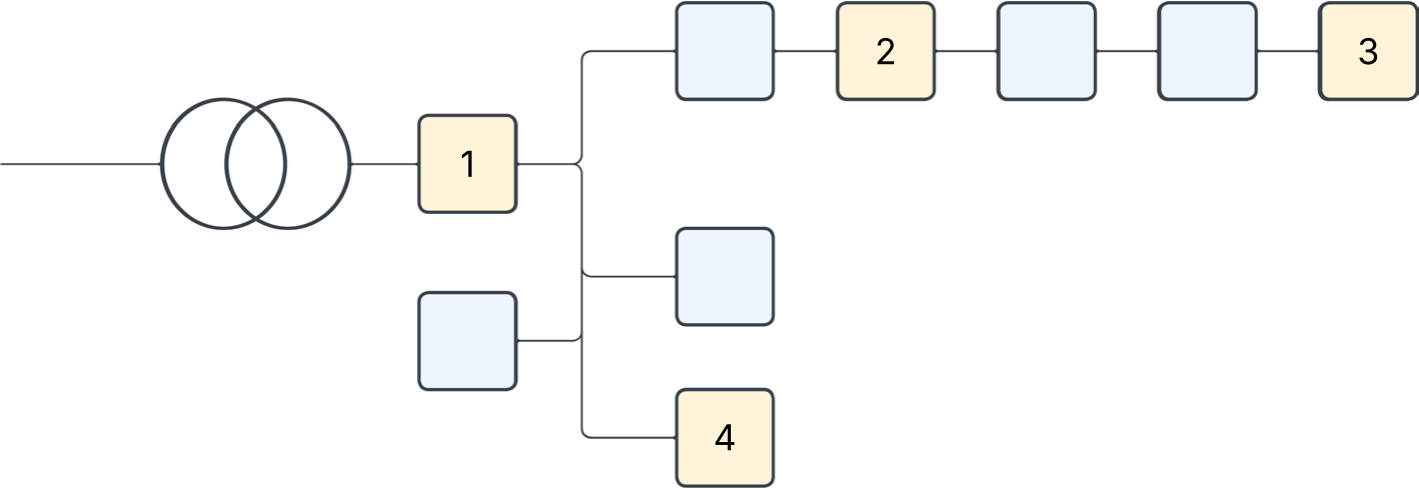
Schematic overview over the rural area real-world case study, featuring the measured nodes (yellow) (1) the transformer station, (2) a farm, (3) a house at the end of the feeder, and (4) a big solar roof, as well as other unmeasured buildings (blue)

The second real-world case study focuses on a rural LV feeder near the village of Markelo in the eastern part of the Netherlands. An overview over the setup is given in Fig. 4.

This setup is also used as a living laboratory in other research (see [77, 78]).

The LV feeder substation supplies five branches, including three additional feeders. One feeder connects to a large solar roof installation, while the others serve farms, houses, and various rural facilities. The feeder is monitored using Phasor Measurement Unit (PMU) sensors provided by Smart State Technology (SST) [79]. These sensors enable detailed tracking of specific connections within the feeder network. The data measured by these PMUs was collected via an internet connection. However, also several unmeasured connections exist along the feeder, situated before, between, and after the measured nodes.

For this case study, we analyse data from four key locations on the feeder: (1) the transformer station, (2) a farm, (3) a house at the end of the feeder, and (4) the solar roof installation. As input for the IDS we created data for all four locations from this living laboratory, one for each of the key points. The data for each contains active (P) and reactive (Q) power measurements, voltage and current measurements, and a time stamp. All measurements are measured in 3 phases. The data from the solar roof installation (4) is the only that does not contain voltage measurements. Importantly, no sensors are installed at the MV side of the transformer supplying this feeder, so only the measurements on the LV side can be accessed. The data is collected at a resolution of one minute. Note that PMUs are capable of recording data at higher frequencies and capturing additional measurements. However, for clarity and consistency in our case studies, we focus on a selected subset of measurements at a coarser temporal resolution. It is worth noting that a one-minute resolution is already high for many grid monitoring applications. We use this granularity here because the data was available and because higher temporal resolution can be valuable for protecting particularly critical or high-value infrastructure within the grid.

## Methodological extension of the IDS

In this section, we present how we adapt the IDS for the two presented real-world case studies. In Section "Requirement adaption for the real-world case studies" dedicated requirements for the two case studies, as well as a new method to fine tune the sensitivity of the IDS in Section "Method for effective threshold calibration for requirements". A discussion of important design decisions taken during the implementation are given in Section "Design decisions and implementation".

### Requirement adaption for the real-world case studies

In this section, we demonstrate how the process-aware IDS (see Section "Background on process-aware IDS" ) is applied to the real-world case studies (see Section "Real-world case studies" ). The IDS comprises different computing units, referred to as monitors, which locally verify predefined sets of requirements against incoming data. The data from all locations of the first case study (three in total) and the second case study (four in total) is assigned to a local monitor (LM), which primarily supervises the respective grid node and its data. As in [24], we include neighbourhood monitors (NMs) between nodes which are located next to each other. Unlike LMs, which focus on local data, NMs oversee connections between two adjacent nodes. In the DSO case study, one NM monitors the connection between MV station 1 and the LV station, while the second supervises the link between both MV stations. For the rural area case study, we implement NMs between (i) the transformer station and the solar roof installation, (ii) the transformer station and the farm, and (iii) between the farm and the house. Furthermore, we introduce a third type of monitor, the neighbourhood plus monitor (NM+) which supervises the entire feeder branch, evaluating so called neighbourhood plus requirements that integrate sensor readings from multiple locations. In the following, we designate all local requirements with an “L” followed by a number, all neighbourhood requirements with an “N” followed by a number, and all neighbourhood plus requirements with an “N+” followed by a number.

To validate sensor readings, we use predefined requirement sets as introduced by Chromik et al. and their extensions (see [19, 20, 23–25]). These requirements ensure, e.g., that each voltage sensor does not exceed a predefined safety threshold. As part of this work, we introduce additional, novel topology-specific requirements for each case study. This allows the IDS to be tailored to the characteristics of each system, enabling a more detailed supervision of critical components such as transformers.

Table 1 summarises the requirements used for the DSO case study. Eight requirements are implemented in total, whereby five are at the local level and three are at the neighbourhood level.

- The local requirements L1, L3, and L5 check whether a sensor exceeds a predefined maximum, whereby requirement L1 enforces a general safety threshold, while L3 and L5 monitor the transformer’s current and voltage limits at both the MV and LV stations.
- Requirement L4 checks for an a unusually low THD,
- L2 ensures that the current measured on the cable remains within expected historical values for a specific type of facility.

The latter two requirements are based on information regarding existing infrastructure, which enables more specific checks to reduce the potential window for undetected attacks.

At the neighbourhood level, we consider the following requirements:

- N1 evaluates whether the measured current is consist on the cable connecting MV1 and MV2, by verifying that the measured transmission loss remains within an expected deviation $\delta$ [24].
- Requirement N2 is based on [25] and analyses the power flow between MV stations. As this case study does not feature measurements for active and reactive power, we assume a power factor of 0.95. While voltage drops naturally occur along transmission lines, this requirement checks whether the calculated and the measured voltages align within a reasonable margin. For this requirement, also information on cable infrastructure is utilized, such as resistance and reactance.
- Requirement N3 calculates the efficiency of the transformer and checks if it is below a set threshold.

**Table 1:** Requirements implemented for the first case study

**Name**	**Requirement**	**Scope**
L1	Safety threshold per sensor is met	Local
L2	Sanity check for specific facility	Local
L3	THD values sanity check	Local
L4	Transformer: safety threshold for current is met	Local
L5	Transformer: safety threshold for voltage is met	Local
N1	Current check on the cable between MV1 & MV2	Neighbourhood
N2	Power flow solving between MV 1 & MV2	Neighbourhood
N3	Transformer efficiency	Neighbourhood

Table 2 summarises the ten requirements implemented for the rural area case study, whereby three requirements rely only on local information, four requirements incorporate neighbourhood data, and the remaining three requirements integrate data from multiple locations at the neighbourhood+ level. Requirement L1, as in the first case study, ensures that sensors do not exceed predefined limits. Requirements L6 to N+1 verify whether calculated voltage or current values exceed expected thresholds. For example, in the local scope, sensor readings from the house are combined with cable impedance data to verify that the expected voltage at Station 4 remains within a reasonable range (requirement L6). Similarly, requirement N5 uses data from the farm and the house to estimate the voltage at Station 3.

At the neighbourhood+ level, requirements N+1 and N+2 integrate readings from multiple locations to perform additional sanity checks. These comprehensive checks reduce the effectiveness of simple attacks, since modifying a single sensor while maintaining plausible values across the network becomes significantly more challenging.

Lastly, requirement N+3 verifies whether voltage drops along multiple cables exceed expected values.

For the second case study values for current and voltage are available for three phases and cable impedance is given as a complex number. Hence we retain this level of detail throughout the calculations to ensure accuracy. This approach accounts for phase shifts, reactance, and other electrical properties, preventing an underestimation of voltage and current. However, when comparing the result of a computation with a threshold for evaluation purposes, the values of the three phases are converted to a single value from the domain of the real numbers. This aligns with standard cable specifications and simplifies interpretation and adjustment for operators.

**Table 2:** Requirements implemented for the second case study

**Name**	**Requirement**	**Scope**
L1	Safety threshold per sensor is met	Local
L6	Sanity check calculated voltage 4	Local
L7	Sanity check calculated voltage 3 (Input only from the House)	Local
N4	Sanity check calculated voltage 6	Neighbourhood
N5	Sanity check calculated voltage 3 (Input from the House and the Farm)	Neighbourhood
N6	Sanity check calculated current 4	Neighbourhood
N7	Sanity check calculated Voltage 1	Neighbourhood
N+1	Sanity check calculated current 1	Neighbourhood plus
N+2	Sanity check calculated combined current 7 and 8	Neighbourhood plus
N+3	Sanity check calculated voltage drop	Neighbourhood plus

### Method for effective threshold calibration for requirements

Requirements L2, L4, N1, N2, N3 from the first case study and all requirements from the second case study except for L1 rely on fixed values, maxima, or minima to determine whether a sensor measurement exceeds a predefined threshold. While some of these thresholds may relate to physical constraints of cable components or standard safety regulations, others are based on a manual or semi-automated configuration by the operator to account for specific characteristics of a grid topology or compensate for known sensor inaccuracies, e.g. caused by poor GPS signalling. In the following, we call the requirements with thresholds related to physical constraints or standard regulations *static* requirements and those who are configured by the operator *parameter-based* requirements.

Previous research (c.f. [19–22, 24, 25]) primarily relied on simulation data and as such inaccuracies often are not included in the research, making threshold calibration less a point of attention. However, when working with real-world data, a careful calibration of those grid-dependant thresholds becomes essential, as they directly affect the accuracy of the IDS. Overly relaxed thresholds may fail to detect attacks, while excessively strict thresholds can generate numerous false positives, leading to unnecessary alerts. Striking an appropriate balance is crucial to prevent both, undetected intrusions and operator alert fatigue.

The proposed threshold calibration method is based on three measures for definitions for a given set of data points $X = \{X_1, X_2, \dots, X_n\}$:

- The Median Absolute Deviation (MAD) for the measurements is defined as:
  1 $$\begin{aligned} \text {MAD} = \text {median} \left(| X_i - \text {median}(X)| \right). \end{aligned}$$
- The relative MAD for one sensor is given by:
  2 $$\begin{aligned} \text {relative MAD for one sensor} = \frac{\text {MAD}}{\left| \text {median}(X) \right| + \epsilon } \end{aligned}$$
  Whereby $\text {median}(X)$ is the median of that dataset and $\epsilon$ is a small constant to avoid division by zero.
- The relative MAD for a set of sensors 1,..., N is calculated by:
  3 $$\begin{aligned} \frac{1}{N} \sum _{i=1}^{N} \text {relative MAD}_i \end{aligned}$$

To support effective threshold calibration, we propose a structured method that allows operators to systematically define threshold sensitivities. We aim to select representative days from the complete dataset for calibration, ensuring that the thresholds are well-suited to the dataset.

1. Extract sensor data relevant to each parameter-based threshold for multiple representative days.
2. Compute the relative Median Absolute Deviation (MAD) for each day to measure variability and ensure unit independence (See Equations 1-3).
3. Identify the days with the highest and lowest relative MAD values within the relevant dataset for each threshold. This ensures that both a highly variable day and a minimally variable day are included in the calibration process, representing the full range of variability within the dataset.
4. Configure the thresholds in the IDS for both selected extreme days to ensure that no false positives are detected.
5. Define upper and lower tolerance boundaries based on these thresholds, i.e. one collection of thresholds representing an upper i.e. more relaxed boundary and one collection of thresholds representing a lower i.e. stricter boundary.
6. Provide the operators with the opportunity to set an adjustable parameter $\alpha \in [0,1]$, to fine-tune a concrete setting of thresholds based on their preference:
  4 $$\begin{aligned} \text {threshold}_i = \text {lowerThreshold}_i + \alpha \cdot (\text {upperThreshold}_i - \text {lowerThreshold}_i) \end{aligned}$$
  Whereby *i* is a specific requirement. Note that, in this way, $\alpha =1$ leads to the upperThreshould and therefore to applying a more relaxed boundary, resulting in fewer false positives. Accordingly, $\alpha =0$ leads to the lowerThreshold being applied, leading to a stricter boundary and prioritizing a more sensitive IDS and the reduction of false negatives.

This method provides an effective threshold calibration, enabling the IDS to maintain high detection accuracy while minimising unnecessary alerts. By allowing operators to adjust sensitivity with the parameter $\alpha$ based on real-world data variability, it ensures adaptability to different grid conditions. The evaluation section (see Section "Evaluation" ) examines the impact of these thresholds in detail.

### Design decisions and implementation

In this section, we discuss key design decisions made during the implementation process. The prototype implementation from [26], which is available open-source on GitLab^1^, serves as foundation for this work. Each distributed component of the IDS (e.g., monitors) is implemented within the OPC-UA framework. Furthermore, to facilitate development and deployment across different hardware platforms monitors are containerized using Docker [80].

We adapted both, the IDS and the replay tool that is developed alongside with the IDS, which remains necessary since the IDS is not tested in real-time directly within a SCADA network. The replay tool uses CSV files containing pre-recorded data along with configuration files as input and then simulates a Modbus server, mimicking real substation servers. The updated implementation is available open-source on GitLab^2^. The configuration format and CSV input structure are adapted to handle real-world data from multiple sources, accounting for varying data resolutions. These modifications improve flexibility and facilitate the integration of future test cases into the IDS.

Three key adaptations are made to the IDS: (1) *Enhanced monitor connectivity and configuration*, (2) *Improved sensor representation*, and (3) *Transition from fixed requirements to a configurable requirement library*.

(1) *Monitor connections:* Previously, each local monitor transmitted its data to a single neighbourhood monitor in a *fire-and-forget* manner. In the updated implementation, local monitors can share data with multiple neighbourhood monitors, depending on their configuration. Additionally, neighbourhood monitors now selectively process data based on the grid topology rather than applying all requirements uniformly to each received dataset. These refinements improve adaptability, allowing the IDS to be tailored to different grid architectures.

**Fig. 5:**
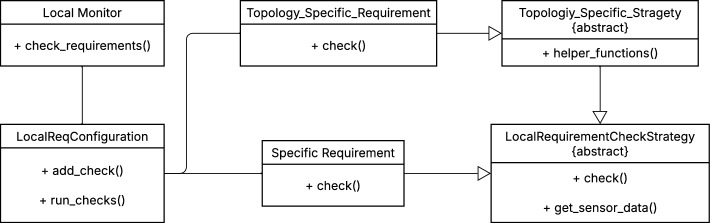
UML class diagram of the new Requirement Library

(2) *Sensor variability:* Originally, the IDS implementation assumed that each sensor measured both single-phase voltage and current. While this assumption is valid for the utilized simulations, it proved to be impractical for real-world case studies, where voltage and current are often measured by separate sensors and additional parameters such as THD or noise levels may also be recorded. To address this, the IDS now treats each sensor as measuring a single independent value. This implies, e.g, that for three-phase voltage measurements each phase is now considered as a separate sensor within the IDS.

(3) *Requirement Library:* The implementation of requirement checks was redesigned to accommodate diverse topologies and incomplete sets of measurements. The original implementation evaluated all test cases with the same predefined set of requirements, assuming the same sensor availability across all grid nodes. This approach, while suitable for simulations, is too rigid for the two considered case studies.

To enhance flexibility, the mechanism for requirement-checking is refactored using the *Strategy Pattern* and the *Open/Closed Principle*. The *Open/Closed Principle* states that software components should be open for extension but closed for modification. Applying this principle to the IDS allows new requirement checks to be introduced without altering existing code. The *Strategy Pattern* enables dynamic selection of different requirement-checking algorithms by encapsulating them in separate, interchangeable classes. Each requirement check is now implemented as an independent class, allowing for modular expansion.

An overview of the newly implemented requirement-checking mechanism is shown in Figure 5. A RequirementChecker in each monitor instantiates a RequirementConfiguration object, which determines the specific set of requirements to be evaluated. The RequirementConfiguration includes a run_check method that executes the selected requirements, while the RequirementChecker itself remains agnostic to the specifics of each check, ensuring a separation of concerns.

All requirement classes inherit from a common abstract base class, which defines an abstract method check that must be implemented for each requirement. This structure ensures encapsulation, modularity, and ease of extension. In addition, some requirements depend on intermediate results shared across multiple checks. To accommodate this, intermediary classes were introduced, such as those handling local monitor evaluations in the second case study. These intermediary classes, also derived from the abstract base class, provide helper functions for reuse across multiple requirement checks, preventing code duplication.

This modular design improves the testability and maintainability of the IDS. Moreover, common requirements, such as those originally defined by Chromik et al. [19, 20, 23], can now easily be reused and configured dynamically for different grid topologies.

## Evaluation

In this section, we evaluate the enhanced IDS that has been adapted for each of the two considered case studies. The evaluation is conducted using real-world data derived from actual grid infrastructure, which cannot be publicly disclosed due to confidentiality and security restrictions. The data of the first case study was recorded by a Dutch DSO obtained at two MV stations. The data of the second case study was recorded on a rural LV feeder near the village of Markelo in the eastern part of the Netherlands. The feeder is monitored using Phasor Measurement Unit (PMU) sensors provided by Smart State Technology (SST)[79]. Key data characteristics, including temporal resolution and measurement types, are detailed in the methodology section (see Section "Real-world case studies" ), and the IDS threshold configurations are provided in the appendix (see Appendix A). We execute various attacks on the real-world case studies. This means that the input files are manipulated according to our attack model (see Section" Attack model" ). Furthermore, we examine which requirements enable the IDS to detect a specific attack.

- A true positive (TP) is an alert triggered by the IDS in response to an actual attack.
- A true negative (TN) occurs when data free of attacks is correctly identified as uncompromised.
- A false positive (FP) is an alert generated by the IDS without an attack.
- A false negative (FN) occurs the IDS fails to recognise manipulated data as compromised, meaning that no alert is issued.

Based on this classification, the *true positive rate* (TPR) and the *true negative rate* (TNR) are defined as follows:

5 $$\begin{aligned} \text {TPR} = \frac{\text {TP}}{\text {TP}+\text {FN}} \end{aligned}$$

6 $$\begin{aligned} \text {TNR} = \frac{\text {TN}}{\text {TN}+\text {FP}} \end{aligned}$$

The TPR is also known as *sensitivity*, while the TNR is referred to as *specificity*. These metrics are widely recognised criteria for assessing the performance of an IDS [81].

To evaluate the effectiveness of the thresholds proposed in Subsection "Method for effective threshold calibration for requirements", we examine the TNR for different requirements that have no attacks in *Scenario 1* for both case studies below. Instead of combining the TNR values from all requirements at one point in time, we suggest averaging the TNR across the various requirements. This method provides a better assessment, ensuring that no single requirement has too much influence on the overall result.

### Real-world case study 1 - DSO

For the first real-world case study, in Section "Scenario 1" we access the number of false positives in the original data (Scenario 1). Next in Section "Scenario 2" we examine a subtle attack on a single location, where false data is injected into six different sensors (Scenario 2). Finally, in Section "Scenario 3", we evaluate the capability of the IDS to detect a replay attack targeting sensors around the transformer (Scenario 3).

#### Scenario 1

**Fig. 6:**
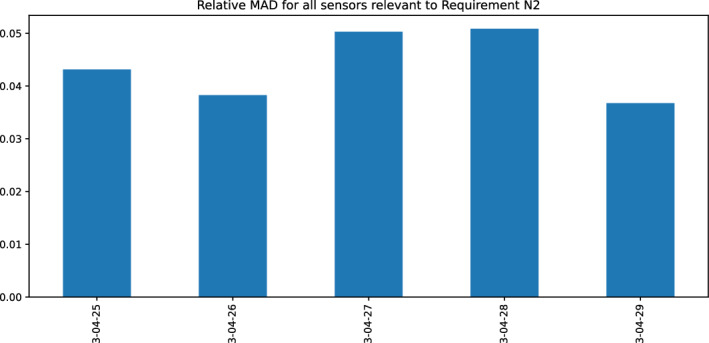
Relative MAD for all sensors incorporated in Requirement N2 for the DSO case study

In this first scenario, we test the impact of the parameter-bases=d thresholds. We calculate the relative MAD (see Equation 3) for all five parameter-based requirements used in the DSO case study over a five-day dataset. As an example, in Figure 6 the relative MAD for all sensors used in Requirement N2 is shown. The highest MAD occurs on 28th April (MAD $\approx$ 0.0508) and the smallest on 26th April (MAD $\approx$ 0.0382).

We configure the thresholds for the parameter-based requirements according to the days with the highest and lowest MAD values (Step 4 of the calibration method as described in Section "Method for effective threshold calibration for requirements " and Equation (3), eliminating false positives on those days. For completeness, the thresholds for the parameter-based requirements are given in Table 11 in the Appendix.

The IDS is then tested on the full dataset with different values for $\alpha \in [0,1]$. The results are summarized in Table 3. The table shows the TNR rate for each requirement with some false positives and the average TNR for these requirements. Herby, a TNR of 1 indicates that no false positives are detected. For $\alpha =1$ three of the configured requirements have a TNR of 1 and the other two only detect few false positives. For $\alpha =0$ four of five requirements still have a TNR larger than 0.95, leading to an average TNR of 0.961. The results clearly indicate that using a smaller $\alpha$ leads to more false positives, which means a lower true negative rate.

**Table 3:** True Negative Rate (TNR) per requirement for the DSO case study, rounded

$\alpha$	TNR AVG	TNR REQ L2	TNR REQ L4	TNR REQ N1	TNR REQ N2	TNR REQ N3
1	0.999	1	1	0.998	0.997	1
0.75	0.989	0.977	0.998	0.995	0.992	0.983
0.5	0.982	0.947	0.998	0.992	0.99	0.983
0.25	0.9768	0.937	0.997	0.982	0.985	0.983
0	0.961	0.914	0.993	0.958	0.965	0.977

#### Scenario 2

Scenario 2 tests the IDS’s ability to detect actual sensor manipulations. In this scenario, 10% of the time steps were chosen at random to be manipulated by an attacker that has access to the network. We hereby focus on the effect of small data manipulations. The attacker only manipulates the data from the MV1 location i.e. the data of the other two location is not changed. To put the severity of this attack into perspective, Table 4 summarizes the relative MAD for each attacked sensor in the MV1 location. Before manipulations, the values of the six attacked sensors have an average relative MAD of 32,19%. Note that, the voltage measurements recorded at this location is measured in kV, leading to a corresponding small percentage of mean average deviation. As part of the attack, the three current sensors and three voltage sensors are altered to either 108% or 92% of their original values, simulating a relatively small yet impactful attack.

**Table 4:** Relative Mean Absolute Deviation (MAD) in % for the six attacked sensors in Scenario 2 of the DSO case study

	AVG	V1	V2	V3	C1	C2	C3
Relative MAD (%)	32.19	0.43	0.45	0.47	107.69	55.55	28.56

In this scenario, the IDS evaluates the attacked datasets using $\alpha =0$ and $\alpha =0.5$. We focus our analysis on $\alpha =0$ and $\alpha =0.5$ instead of $\alpha =1$, to examine a more sensitive IDS in relation to the attacks. The results are summarised in Table 5. Unlike Scenario 1, where we focused on the true negative rate (TNR), which measures false positives relative to true negatives, for this attack scenario we evaluate the true positive rate (TPR), which accounts for false negatives relative to true positives.

The IDS is able to detect all attacks in both setups in case all requirements were considered, resulting in a TPR of 1. However, the detection capability varies based on the considered requirement. Notably, both L1 (54) and L5 (60) show high detection rates, while the neighbourhood requirements have lower detection rates. These two local requirements test if sensor values exceed a general maximum for that sensor (L1) or a specific voltage maximum set by the transformer (L5). Therefore, a change of 8% compared to the original value almost always exceeded these limits, triggering an alert. In contrast, the lower detection rates for the neighbourhood requirements could be expected because the thresholds defined in Scenario 1 were very low, allowing small changes to go undetected.

Note that currently, no specific action is implemented in case the power flow equation solving in requirement N2 does not converge. However, repeated failures could indicate potential attacks or system instability and integrating such cases into the IDS logic may improve robustness in the future.

**Table 5:** Number of alerts detected by each requirements in Scenario 2 of the DSO case study

$\alpha$	TPR	# unique alerts	L1	L2	L5	N1	N2	N3
0	1	60	54	10	60	2	7	5
0.5	1	60	54	10	60	0	4	1

#### Scenario 3

In Scenario 3, we investigate replay attacks focusing on a particularly vulnerable component of our case study, the transformer. To create the attack, we now replace 10% of the values of the selected sensors with historical data from the corresponding sensor. We implement the attack in two ways: For the first attack, we replace the values of the three voltage sensors on the LV side of the transformer and for the second attack, we replace the values of the current sensors on the LV side and the voltage sensors on the MV side of the transformer.

Table 6 summarizes the results for these two attacks. We focus our discussion on requirement N3, which evaluates transformer efficiency. Since the attack merely replays historical data, it cannot detected by other transformer requirements that monitor whether maximum values are exceeded. In the first attack, requirement N3 has a TPR of 0.3103. For the second attack, a TPR of 0.339 is achieved. These low detection rate can be partly attributed to the conservative threshold for transformer efficiency set in Scenario 1. This is due to the fact that in the available datasets, the measurements from the two MV stations were recorded every ten minutes starting at 12:00, while the LV station data was recorded every ten minutes starting at 12:02. This time shift, along with other possible inaccuracies or sensor-specific differences, results in a very low threshold for Requirement N3, as the requirement combines data from MV 1 and LV 1 to evaluate transformer efficiency. This effect is seen for all values of $\alpha$, but it is particularly noticeable for the threshold that corresponds to $\alpha =0$.

Note that manipulations on the MV side may also be detected by other requirements, since voltage is used as an input. In contrast, this is not the case for the LV side, as we currently do not have additional requirements incorporating voltage or current, aside from those that check against maximum or minimum thresholds.

Additionally, due to the limited dataset (five days), the shuffled historical data often closely resembles the actual measurements. For instance, in the first attack, there were 22 cases where the difference between the original and the new voltage values was less than 5 V. In the second attack, 80 instances were recorded where the LV current differed by 1 A or less, and 45 instances showed a difference of 0.5 A or less. Similarly, for the MV voltage, 29 cases had a difference of 5 V or less. In such instances, the minimal difference between the actual and manipulated values raises questions about the overall impact of the attack even if it goes undetected.

**Table 6:** Summary of attack detection performance of Requirement N3 in Scenario 3 of the DSO case study

**Variant**	**Total Attacks**	**Detected by N3**	**TPR**
1: Only LV V	58	18	0.3103
2: LV C and MV V	59	20	0.3390

### Real-world case study 2 - rural area

Similar to the DSO real-world case study, we start by accessing the number of false positives in the original data (see Section "Scenario 1" ). Next, we evaluate the capability of the IDS to detect a subtle attack targeting the data of a single location (see Section "Scenario 2" ) and in Section "Scenario 3", we examine a sequential attack.

#### Scenario 1

To further evaluate the proposed method for threshold configuration, we test the IDS using the dataset from the rural area case study, which spans five and a half months and by that is significantly larger. The approach is consistent with Scenario 1 from the DSO case study, which focuses on evaluating the parameter-based thresholds. Given the large dataset, we selected two full months, December and April, to calculate the MAD values for all parameter-based requirements.

The IDS is then calibrated using the days with the highest and lowest relative MAD values from December (see (3)), and the resulting thresholds are applied to evaluate the entire month of January. Similarly, the days with the highest and lowest relative MAD values from April are used to configure the IDS for evaluating May. Additionally, we combine the threshold values from December and April and apply them to January and May under two parameter settings: $\alpha =0$ (minimizing false negatives) and $\alpha =1$ (minimizing false positives). For completeness, we summarize these operator defined threshold in Table 12 in the Appendix.

Unlike the first case study, the parameter-based thresholds in this analysis represent values which are not directly measured but instead calculated by the IDS, for example a maximum voltage at a grid point. Although strict infrastructure limits exist for such voltages and are dictated by factors like cable capacity, we opt to configure the thresholds based on the calibration method rather than using these predefined limits. When configuring the IDS using datasets (e.g. the one from December) some parameter-based maxima are set lower than similar voltage limits for directly measured values. As a result, this leads to more false positives, where voltages exceeded the parameter-based thresholds but remained within actual infrastructure limits. Despite this, we argue that calculating these maxima remains valuable because they account for factors such as poor GPS signals, time shifts, and sensor-specific characteristics. However, in practice, adapting the threshold accordingly when they are lower than expected infrastructure limits could help reduce false positives.

The results of this extended evaluation are summarised in Table 7. For $\alpha =1$ average the TNR over all requirements ranges between 0.954 and 1 in the combined case. It can be seen that the TNR over the different parameter-based requirements varies. In general, the TNR for $\alpha =1$ is larger or equal to the ones for $\alpha =0$, as expected. Interestingly, calibrating the thresholds for January based on December data performs worse than defining the thresholds for May based on April data. This may indicate that the data from April is more representative for May than the December data is for January.

As indicated in Table 12, the thresholds based on December data are smaller than those based on April data. Accordingly, when the combined thresholds are used, the higher April threshold lead to higher threshold for $\alpha =1$ and the lower December thresholds lead to a lower threshold for $\alpha =0$. As a result, the IDS becomes more robust in case $\alpha =1$ is used and more sensitive in case $\alpha =0$ is applied.

**Table 7:** True Negative Rate analysis for the rural area case study, TNRs are rounded

Setup	TNR AVG	TNR L6	TNR L7	TNR N4	TNR N5	TNR N6	TNR N7	TNR N+1	TNR N+2	TNR N+3
Dec $\rightarrow$ Jan, $\alpha =$ 1	0.954	0.942	0.932	0.996	0.989	0.964	0.959	0.946	0.915	0.941
Dec $\rightarrow$ Jan, $\alpha =$ 0	0.941	0.942	0.932	0.996	0.989	0.940	0.959	0.941	0.830	0.936
April $\rightarrow$ May, $\alpha =$ 1	0.987	0.984	0.980	1.000	0.998	0.998	1.000	0.999	0.938	0.989
April $\rightarrow$ May, $\alpha =$ 0	0.960	0.966	0.962	1.000	0.997	0.988	0.998	0.994	0.854	0.881
Dec&April $\rightarrow$ Jan, $\alpha =$ 1	1.000	1.000	1.000	1.000	1.000	0.998	1.000	1.000	1.000	1.000
Dec&April $\rightarrow$ Jan, $\alpha =$ 0	0.941	0.942	0.932	0.996	0.989	0.940	0.959	0.941	0.830	0.936
Dec&April $\rightarrow$ Mai, $\alpha =$ 1	0.987	0.984	0.980	1.000	0.998	0.998	1.000	0.999	0.938	0.989
Dec&April $\rightarrow$ Mai, $\alpha =$ 0	0.909	0.863	0.857	1.000	0.948	0.925	0.956	0.932	0.832	0.869

#### Scenario 2

Similar to Scenario 2 in the DSO case study, we perform attacks on 10% of all time steps in May by randomly altering the values of the three voltage and three current sensors in the data collected at the Farm location. The data in the other locations do not get attacked. Again, to put the severity of this attack into perspective, Table 8 summarizes the relative MAD for each altered sensor in the original location data. The values of the affected sensors have an average relative MAD of 35%. Again, the deviation in the measured voltage is much smaller than the deviation in the measured current. We randomly change the value of the attacked sensors to either 108% or 92% of their original value leading to an alteration by ± 8% respectively. This attack simulates an attacker with network access who tests the impact of subtle data manipulations, which, while small, are not entirely insignificant.

**Table 8:** Relative Mean Absolute Deviation (MAD) in % for the six attacked sensors in Scenario 2 of the rural area case study

	AVG	V1	V2	V3	C1	C2	C3
Relative MAD (%)	35	0.94	1	0.87	58.49	72.34	76.32

We let the IDS detect the introduced manipulations using $\alpha =0$ and $\alpha =0.5$. The results for Scenario 1 are summarised in Table 9. For clarity, requirements that did not use data from the manipulated sensors are not included in the table.

In both cases, the IDS detects all manipulated time steps, achieving a TPR of 1. However, as in Scenario 1 of the DSO case study, the table shows that the requirements detected the manipulations to varying degrees. The local requirement for checking whether measured values exceed a given maximum (L1) is effective in this scenario as well. For the other requirements, it is noticeable that some requirements (N6, N7, N+1, N+3) are also highly effective, while requirements N5 and N+2 detect fewer manipulations. However, for these two requirements, there is a clear difference in detection rates between $\alpha =0$ and $\alpha =0.5$, highlighting the importance of the operator’s choice of $\alpha$.

**Table 9:** Number of alerts detected by each requirements in Scenario 2 of the rural area case study

Setup	TPR	# unique alerts	L1	N5	N6	N7	N+1	N+2	N+3
$\alpha =0$	1	437	351	192	437	237	437	394	437
$\alpha =0.5$	1	437	351	125	437	237	437	134	437

#### Scenario 3

In Scenario 3 of the rural area case study, we examine a sequential attack. Similar to Scenario 1, we manipulate 10% of the data from the Farm location, but this time the manipulation is introduced gradually to simulate a sequential attack. At each attack step, the applied shift is proportional to the ratio of the attack step index over the total number of available time steps. Given the large number of available time steps, the shift applied in this scenario is much smaller compared to Scenario 1, where a fixed percentage based on the original value was used. We evaluate this scenario for both $\alpha = 0$ and $\alpha = 0.5$. Table 10 summarizes the detection steps for each requirement, indicating when each first identifies the attack. Since the manipulated sensors are not the only inputs for each requirement, the time of attack detection can vary. However, it can be seen that the earliest detections are similar for both values of $\alpha$, or are very close. This suggests that, despite the smaller manipulations, the attacks in the data are easily identifiable as outliers, regardless of $\alpha$ value.

It is important to note that not all subsequent attacks, after the initial detection as noted in Table 10, are necessarily detected by the corresponding requirement. This is because the other input variables for each requirement may still allow more room for the attack before the detection threshold is reached, even with the increased manipulation.

**Table 10:** Earliest detected time step with an attack per severity level for each requirement

$\alpha$	L6	L7	N5	N6	N7	N+1	N+2	N+3
0	72	72	529	74	1080	62	63	215
0.5	75	75	529	74	1080	62	67	215

## Discussion

In this section, we discuss the results and insights from Section"Evaluation"from a broader perspective, considering their implications for the overall approach.

The evaluation of the IDS across multiple scenarios and two real-world case studies demonstrates that the IDS can effectively detect attacks using existing measurement points in the grid. This suggests that leveraging available data could enhance cybersecurity without requiring significant changes in infrastructure. However, the results also highlight key factors influencing IDS performance, such as the calibration of parameter-based thresholds, the resulting trade-off between false positives and false negatives, and the impact of data availability on detection accuracy.

In both case studies, the IDS is able to successfully detect all manipulated time steps in Scenario 2, thereby achieving a true positive rate (TPR) of 1. However, detection effectiveness varies for different requirements. Where local requirements (e.g. like L1 and L4 in the DSO case study checking for maximum or minimum sensor values) performed well for the performed attacks, the neighbourhood-based requirements did not necessarily catch the attacks in the same degree. This does not mean, that the neighbourhood requirements are less valuable than the local requirements, but that they detect attacks differently. For the IDS to successfully detect various kinds of attacks, it is important to utilize multiple requirements which have a different focus, to minimize the risk of attacks remaining unnoticed. However, when incorporating additional requirements, it is crucial to ensure that the evaluation process remains (near) real-time capable, which also depends on the hardware used for the various IDS monitors. An initial discussion of this consideration is provided in [26].

Another challenge is the influence of time shifts and sensor inaccuracies on the threshold calibration as defined in the proposed method. For example, the configured threshold for Requirement N3 this is configured really low due a time shift and other possible inaccuracies or sensor-specific differences in the datasets. The measurements from the two MV stations were recorded every ten minutes starting at 12:00, while the LV station data was recorded every ten minutes starting at 12:02. This effect impacts the first case study, particularly in Scenario 3, where it is most evident. In the second case study, brief GPS signal loss or deviations from the nominal 50 Hz grid frequency may affect the accuracy of data recorded by PMU devices. A larger dataset and improved synchronization between sensors can help to refine thresholds and enhance the detection accuracy. Further refinements, such as incorporating additional dates in Step 3 of the proposed calibration method beyond the highest and lowest MAD days, may also improve threshold calibration. Alternatively, operators may set thresholds higher than those suggested by the calibration method while carefully considering the trade-off with false positive rates. This underscores the importance of threshold selection, as small attack-induced deviations may remain undetected if they fall within normal variations of data.

Although some of the original requirements defined by Chromik et al. have not be implemented due to missing data points, the shift to more topology-aware and adaptable requirements provided valuable insights. The inclusion of additional measurement points in the grid would likely enhance both threshold calibration and detection performance, by reducing the risk of attacks being not undetected. These findings may help to identify key locations where extra measurement points would be most beneficial, improving IDS effectiveness and by that improving future infrastructure planning.

## Conclusion

In this paper, we introduced an improved IDS tailored for smart grid security. Our contributions include a novel approach for defining IDS requirements, an effective calibration method for setting detection thresholds to balance false positives and false negatives, and a flexible IDS implementation adaptable to different grid topologies. We demonstrated the system’s applicability through two real-world case studies, one on the LV grid and another on the transition between Low and MV grids. In six different scenarios we showed that the IDS can successfully detect attacks based on the chosen attack model.

This solution is particularly relevant for DSOs, as our research focuses on the grid level spanning MV to LV stations and local feeders, that are typically under DSO management. Currently, the system sends alarms to the DSO, who is responsible for taking appropriate action, as implementing fully automated responses remains a highly complex challenge. Introducing multiple alarm levels, such as notice, warning, emergency, could enhance operational response in future work.

Despite promising results, challenges persist, particularly in addressing the balance between false positives and negatives. The case studies were limited in scope, both geographically and temporally, and lack certain sensor data necessary for more specific requirements. Consequently, further validation with larger and more diverse datasets is required to fully assess the scalability and performance of the system.

Further improvements will focus on optimizing detection thresholds, integrating historical data for better intrusion detection, and refining sensor placement for enhanced security while being cost efficient. Where this study takes a step toward practical IDS implementation, ongoing development and testing under varied conditions are essential to maximize its reliability and effectiveness. Future work may also focus on real-time monitoring, including direct network traffic analysis and rapid response to threats, enabling the IDS to function alongside operational grid infrastructure. For that, operator behaviour and unusual grid behaviour in case of e.g. maintenance needs to be integrated appropriately.

## Data Availability

The source code of the IDS implementation and the supporting replay tool is available on https://gitlab.utwente.nl/vmenzel/ids_realworld_scenarios. The used data sets and configuration files are confidential, the evaluation files of the six scenarios are available along with the source code on GitLab.

## Appendix A −Exemplary parameter-based thresholds

See Appendix Tables 11, 12.

**Table 11:** Parameter-based thresholds configured according to the calibration method for the DSO case study

$\alpha$	REQ L2	REQ L4	REQ N1	REQ N2	REQ N3
1	0.234	0.71	0.38	1.72	2
0	0.234	0.67	0.12	1.55	44

**Table 12:** Parameter-based threshold configured according to the calibration method for the rural area case study

$\alpha$	L6	L7	N4	N5	N6	N7	N+1	N+2	N+3
1 DEC	239	239	240	240	118	240	392	2407	64
0 DEC	239	239	240	240	83	240	370	1711	62
1 APR	243	243	242	244	198	247	759	2948	127
0 APR	242	242	240	243	147	245	641	1927	68
1 TOTAL	243	243	242	244	198	247	759	2948	127
0 TOTAL	239	239	240	240	83	240	370	1711	62

## Footnotes

1 The source code is available at https://gitlab.utwente.nl/vmenzel/csr-prototype-distributed-ids.

2 The source code is available at https://gitlab.utwente.nl/vmenzel/ids_realworld_scenarios.
